# Incorporating adjustments for variability in control group response rates in network meta-analysis: a case study of biologics for rheumatoid arthritis

**DOI:** 10.1186/s12874-019-0837-2

**Published:** 2019-10-16

**Authors:** Chris Cameron, Abhishek Varu, Arthur Lau, Mahdi Gharaibeh, Marcelo Paulino, Raina Rogoza

**Affiliations:** 1 Data Analytics & Evidence Synthesis, Cornerstone Research Group, Inc., Burlington, ON Canada; 2 Division of Rheumatology, McMaster University, Hamilton, ON Canada; 3 Amgen Inc., Thousand Oaks, CA USA; 4 Amgen Canada, Inc., Mississauga, ON Canada

**Keywords:** Systematic review, Network meta-analysis, Rheumatoid arthritis, Biologics, Statistical methods

## Abstract

**Background:**

The importance of adjusting for cross-study heterogeneity in control group response rates when conducting network meta-analyses (NMA) was demonstrated using a case study involving a comparison of biologics for the treatment of moderate-to-severe rheumatoid arthritis.

**Methods:**

Bayesian NMAs were conducted for American College of Rheumatology (ACR) 50 treatment response based upon a set of randomized controlled trials (RCTs) identified by a recently completed systematic review of the literature. In addition to the performance of an unadjusted NMA, a model adjusting for cross-study heterogeneity of control group response rates using meta-regression was fit to the data. Model fit was evaluated, and findings from both analyses were compared with regard to clinical interpretations.

**Results:**

ACR 50 response data from a total of 51 RCTs and 16,223 patients were analyzed. Inspection of cross-study variability in control group response rates identified considerable differences between studies. NMA incorporating adjustment for this variability was associated with an average change of 38.1% in the magnitude of the ORs between treatment comparisons, and over 64% of the odds ratio changed by 15% or more. Important changes in the clinical interpretations drawn from treatment comparisons were identified with this improved modeling approach.

**Conclusions:**

In comparing biologics for moderate to severe rheumatoid arthritis, failure to adjust for cross-trial differences in the control arm response rates in NMA can lead to biased estimates of comparative efficacy between treatments.

## Background

During the past decade, network meta-analyses (NMA) have become increasingly common in healthcare research [1, 2]. Applications of NMA have grown in frequency and popularity and can inform the comparison of multiple interventions which may not have been compared directly in head-to-head clinical trials [3–5]. In practice, when undertaking an NMA, researchers must pay careful attention to the extent of variability between studies in terms of both study design and patient characteristics (henceforth referred to as ‘heterogeneity’) to establish the appropriateness of integrating the results from multiple studies in NMA [6, 7]. When well performed, NMAs allow for decision-making in scenarios where direct comparisons of interventions (in the context of clinical trials) are unavailable, however, the end users of such analyses must be made aware of the potential limitations that can emerge if cross-trial heterogeneity is present and is not formally addressed. In such analyses, there is a greater risk of drawing misleading interpretations from the findings around treatment effects [6, 7] and potentially impacting clinical decision-making. Past research has demonstrated how adjusting for cross-trial heterogeneity can potentially play an important role in the validity of meta-analysis and NMAs. For example, Salanti et al. [8], previously demonstrated in NMA-based comparisons of interventions to prevent dental caries that magnitudes of treatment effect, as well as the rank ordering of treatments, were altered when accounting for differences in clinically relevant covariates such as baseline mean caries level [8]. Therefore, it is important to demonstrate the need for assessing systematic differences in treatment effect modifiers across comparisons when conducting an NMA for healthcare decision-makers and researchers. Variability in control group response rate between interventions and studies within NMA can inflate relative estimates of treatment effect for those interventions with values lower than the overall average while biasing against those interventions with higher response rates. Given the common challenge of access to sufficiently large numbers of studies for meta-regression analysis and lack of reporting of many characteristics, [9] the availability of a characteristic such as control group response rate which can indirectly account for variability in multiple measures can be of considerable value.

Comparison of interventions for moderate to severe rheumatoid arthritis (RA) represents one of the most heavily studied therapeutic areas in terms of past applications of NMA, with a total of 28 published between 2003 and 2014 [10]. While well-established methods guidance for NMA has previously noted the importance of incorporation of adjustments for between-study variability in control group risk [6], follow-through on this recommendation has been varied. In 2017, a clinical review reported by the Institute for Clinical and Economic Review (ICER) incorporated adjustments for control group risk in NMAs evaluating the effects of targeted immune modulators for RA [11], as have some other past reviews. Conversely, a number of other NMAs in this clinical area have failed to do so, including the original analysis from which our data was abstracted [12]. Discordance in findings between these reviews is apparent in terms of the interventions that were concluded to be associated with greater extents of clinical benefit. Thus, there remains a need to assess the importance of adjustments for cross-study heterogeneity in control group response rate when comparing interventions for moderate to severe RA to re-affirm for researchers the importance of this inclusion in their systematic review methods when planning future research.

## Methods

An overview of the approach taken to establish the evidence base for the NMAs in a case study is provided, including selection criteria and approaches for the synthesis of the evidence (approaches for the fitting of both unadjusted and adjusted models are noted). Graphical approaches used to establish the existence of heterogeneity between studies and to summarize changes in treatment effects achieved using unadjusted and adjusted models are also presented, and subsequent discussion is focused upon contextualizing the importance of accounting for cross-study heterogeneity when comparing interventions for moderate to severe RA.

### Case study: network meta-analyses of biologic therapies for moderate-to-severe rheumatoid arthritis

**Fig. 1:**
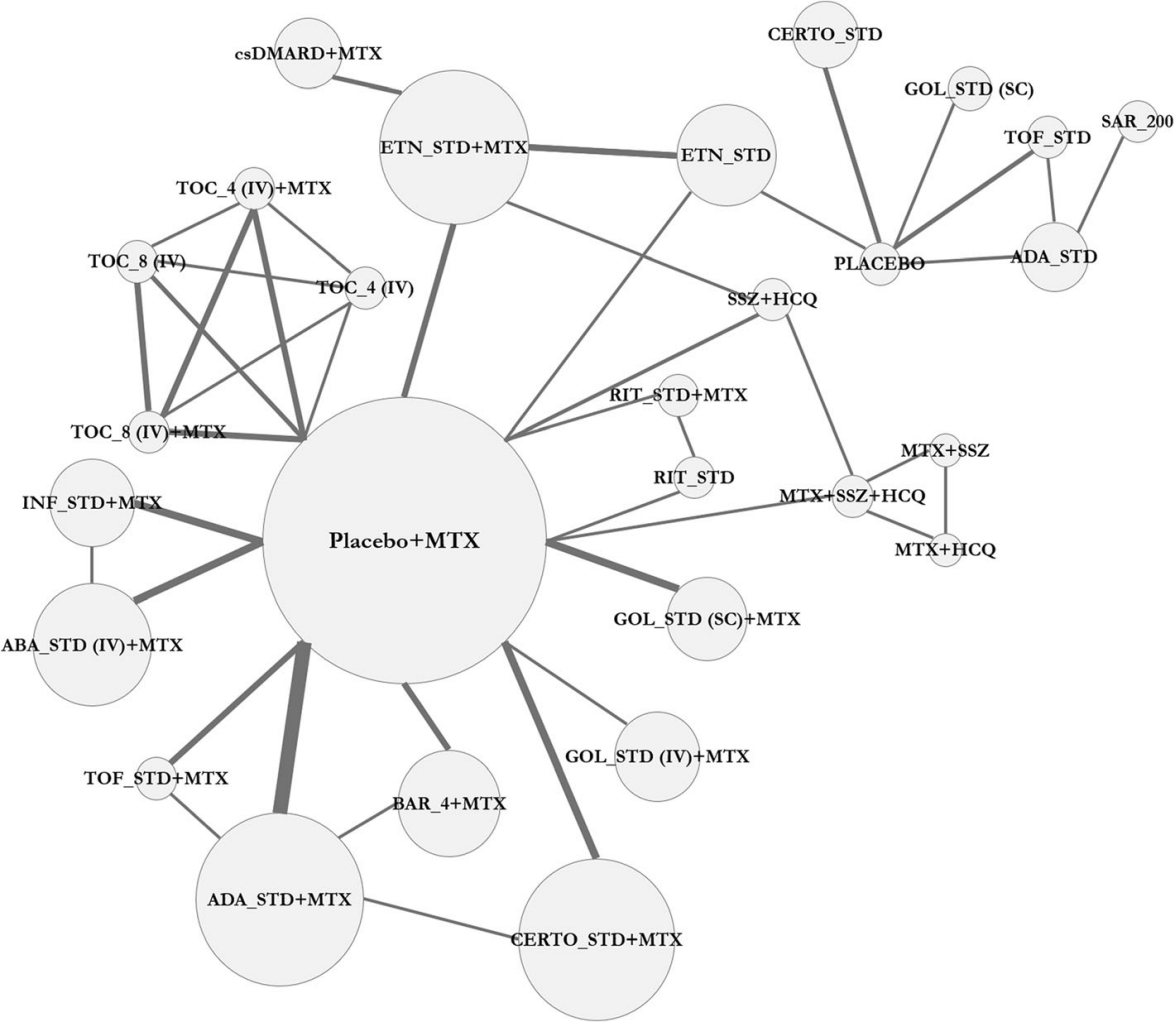
Evidence Network Comparing Biologics for Moderate to Severe Rheumatoid Arthritis, ACR50 Response. Treatment nodes are sized to proportionally reflect the numbers of patients randomized to each intervention, while the line thickness of edges joining nodes are asized to propotionally reflect the numbers of studies informing each comparison. Legend: ABA = abatacept; ADA = adalimumab; BAR_4 = 4 mg baricitinib; CERTO = certolizumab pegol; csDMARD = conventional synthetic disease-modifying anti-rheumatic drug; ETN = etanercept; GOL = golimumab; INF = infliximab; IV = intravenous; MTX = methotrexate; RIT = rituximab; SAR_200 = 200 mg sarilumab; SC = subcutaneous; SSZ = sulfasalazine; STD = standard dose; TOC_4 = tocilizumab 4 mg/kg; TOC_8 = 8 mg/kg tocilizumab; TOF = tofacitinib

To illustrate the importance of adjusting for cross-study heterogeneity in RA, we present an illustration based on an evidence base derived from a recent Technology Review of interventions from the Canadian Agency for Drugs and Technologies in Health (CADTH) for moderate to severe RA [13]. We focus on an example employing innovator biologic interventions in the main text of this report, while further analyses adding consideration of biosimilars are presented in the Additional file 1. Outcome data for this illustration were compiled through inspection of the review’s listing of included studies [13] and subsequent data collection from the trial articles by the research team of this report. Approaches for both the inspection of studies for clinical heterogeneity as well as the performance of NMAs using unadjusted and adjusted models used established models recommended by the National Institute for Health and Care Excellence [6, 7] (NICE; additional modeling details are described below). A total of 51 RCTs (Additional file 1: Appendix 1) (*n* = 16,223 patients) were included for NMA of the ACR 50 (American College of Rheumatology 50) response outcome, a commonly assessed binary outcome measure which captures the proportion of patients achieving 50% or greater improvement in severity of disease from study baseline. This score is a composite of both clinical and laboratory parameters used in the assessment of disease activity. The network diagram presented in Fig. 1 provides an overview of the evidence base available for this outcome measure. The network consisted of many comparisons of biologic interventions against placebo, while a smaller number of head-to-head comparisons were also present. In total, 166 treatment arms were included in the evidence base for ACR 50.

### Statistical methods for unadjusted NMAs of ACR 50 response

To inform comparisons between biologics, unadjusted Bayesian random effect (RE) NMAs using the logit link and binomial likelihood were conducted using R Software (Version 3.5.1, The R Foundation for Statistical Computing) and WinBUGS software (version 1.4.3, MRC Biostatistics Unit, United Kingdom) in accordance with recommendations and statistical code made available by NICE that adjusts for correlation in multi-arm trials [7]; RE models were chosen as the focus of this report given the heterogeneity amongst studies as well as measures of model fit. Vague prior distributions for treatment effects (Normal with mean 0 and precision 0.0001) in both models were used. Odds ratios (ORs) with 95% credible intervals (CrIs) were estimated to capture pairwise comparisons between all interventions (including both biologics and placebo). Surface Under the Cumulative Ranking (SUCRA) curve measures were also estimated to provide the probability of a treatment ranking highly. SUCRA values range from 0 to 100%, with values closer to 100% representing treatments with more favorable rankings for ACR50. These values can be informative for readers in terms of providing an overview of the treatment hierarchy for an outcome of interest. All NMAs were carried out using three sets of starting values and were based on sampling of 40,000 iterations including burn in. Evaluation of model convergence was informed by inspection of trace plots, Gelman-Rubin plots, and Monte Carlo standard error of parameter estimates from the Markov Chain Monte Carlo (MCMC) analysis.

### Evaluating cross-study heterogeneity in control group response rates

**Fig. 2:**
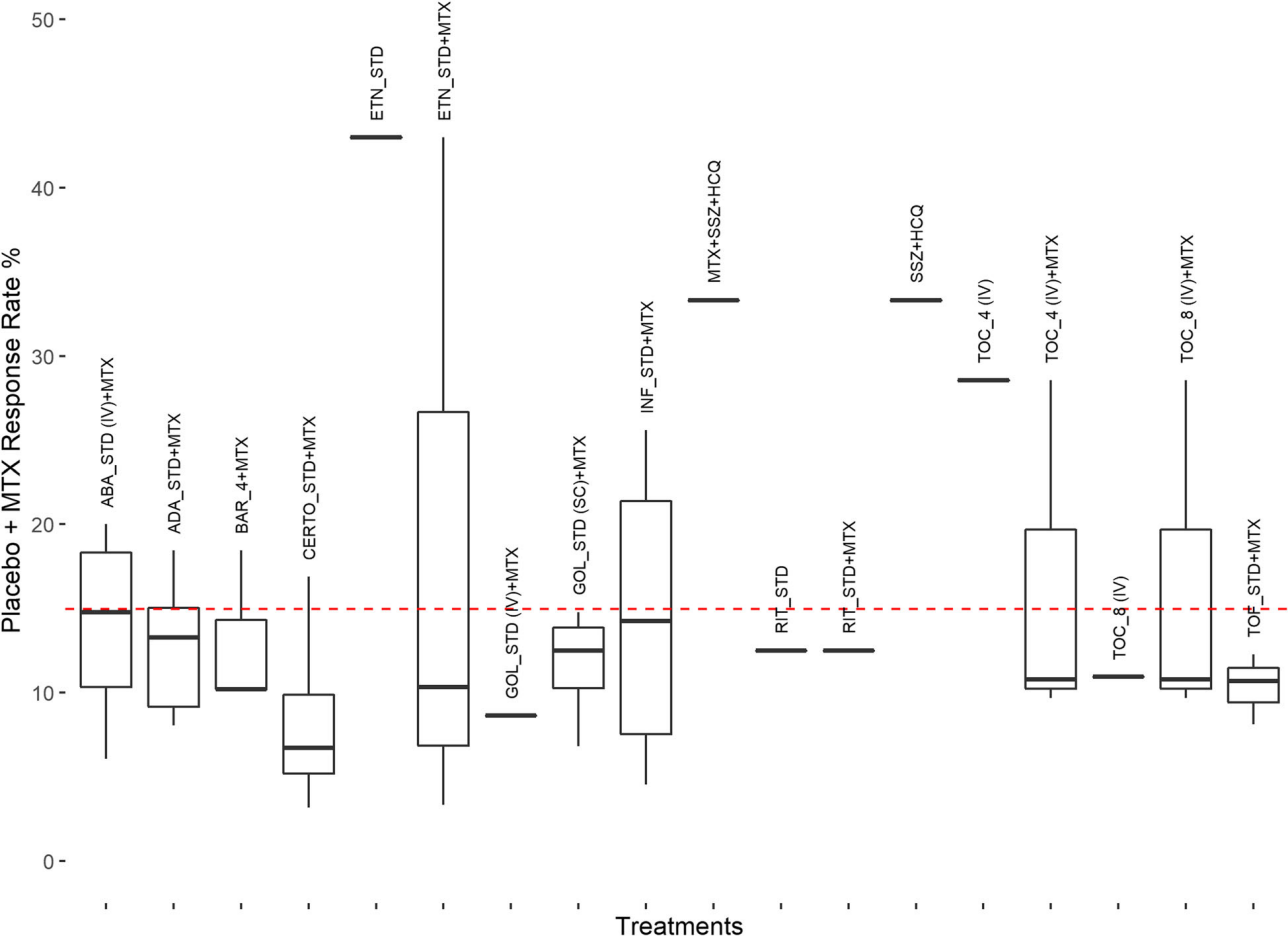
ACR50 response rate of control group, placebo + MTX (ie, MTX) across interventions and studies. A boxplot summary of the control group response rates, by intervention, is shown. Interventions associated with control group response rates above the average line may have unadjusted NMA results biased against them, while interventions with control group response rates below the average line may have unadjusted NMA results biased in their favor. Legend: ABA = abatacept; ABP501 = biosimilar adalimumab; ADA = adalimumab; ANBAI = AnBaiNuo (biosimilar adalimumab); BAR_4 = 4 mg baricitinib; CERTO = certolizumab pegol; csDMARD = conventional synthetic disease-modifying anti-rheumatic drug; CT-P13 = biosimilar of infliximab; ETN = etanercept; GOL = golimumab; HCQ = hydroxychloroquine; HD203 = etanercept biosimilar; INF = infliximab; IV = intravenous; MTX = methotrexate; RIT = rituximab; SAR_200 = 200 mg sarilumab; SB2 = biosimilar infliximab 3 mg/kg; SB4 = biosimilar etanercept 50 mg; SB5 = biosimilar adalimumab; SC = subcutaneous; SSZ = sulfasalazine; STD = standard dose; TOC_4 = tocilizumab 4 mg/kg; TOC_8 = 8 mg/kg tocilizumab; TOF = tofacitinib; ZRC-3197 = biosimilar of adalimumab

Cross-study heterogeneity has been identified in past literature addressing comparisons of biologic interventions for RA [6, 13]. Of particular interest in this methodologic exercise was variability across studies in control group response rate (commonly referred as baseline risk adjustment [6]), a measure which is known to be a proxy for cross-study variability in multiple confounders (both measured and unmeasured) and which has previously been cited as a vital adjustment factor for NMAs of interventions for RA [6]. We generated a box plot of control group response rates (Fig. 2) to identify differences in response rate between intervention groups.

### Statistical methods for adjusted NMAs of ACR 50 response

Bayesian random effect (RE) NMAs were conducted using R Software (Version 3.5.1, The R Foundation for Statistical Computing) and WinBUGS software (version 1.4.3, MRC Biostatistics Unit, United Kingdom) in accordance with recommendations and statistical code made available by NICE TSD to conduct a meta-regression model to adjust for baseline-risk [6]. To assess whether the meta-regression model was better for analysis than the unadjusted model (and thus more suitable for use in drawing clinical interpretations), guidance from the NICE Decision Support Unit (DSU) Technical Support Documents (TSD) was used [6]. This included establishing whether the regression coefficient was associated with a 95% CrI which excluded 0 and whether the between-study standard deviation parameter (and its 95% CrI) was reduced in magnitude; the deviance information criterion (DIC) and the posterior residual deviance were also assessed. As recommended by guidance from the NICE TSD series, decisions about model choice were focused upon all of the above information as opposed to DIC alone, which can be unreliable for such decisions [6].

## Results

### Assessment of variability in control group response rates and relationship with treatment effect

**Fig. 3:**
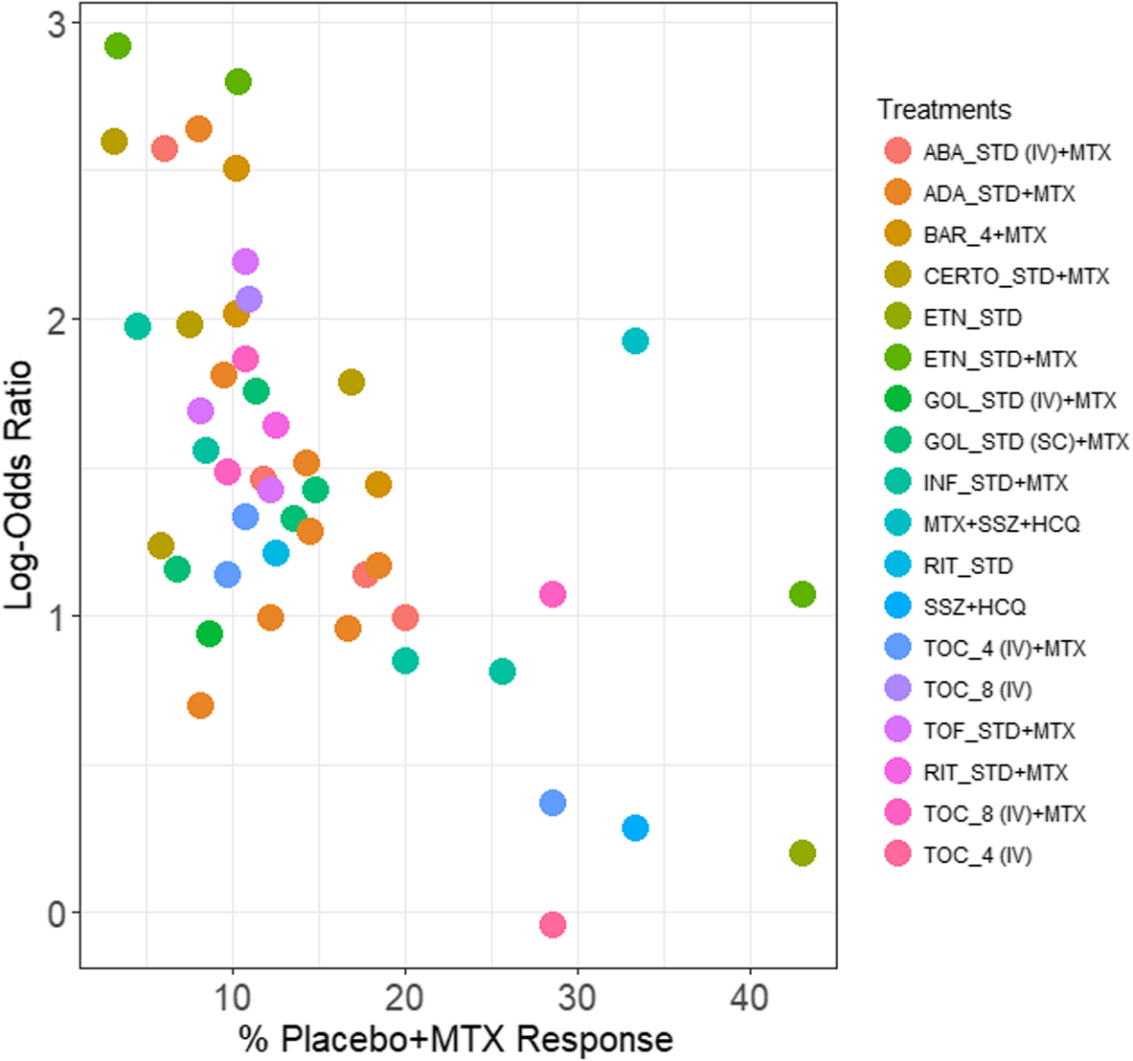
Scatterplot of placebo response rates versus log (OR) for ACR 50 response. A scatterplot of the natural log of each study’s treatment effect against its corresponding control group response rate. Points are color-coded by intervention. A clear relationship exists between % control group response and treatment effect, wherein lower response rates are associated with larger treatment effects. Legend: ABA = abatacept; ADA = adalimumab; BAR_4 = 4 mg baricitinib; CERTO = certolizumab pegol; csDMARD = conventional synthetic disease-modifying anti-rheumatic drug; ETN = etanercept; GOL = golimumab; INF = infliximab; IV = intravenous; MTX = methotrexate; RIT = rituximab; SAR_200 = 200 mg sarilumab; SC = subcutaneous; SSZ = sulfasalazine; STD = standard dose; TOC_4 = tocilizumab 4 mg/kg; TOC_8 = 8 mg/kg tocilizumab; TOF = tofacitinib

Inspection of the bar chart in Fig. 2 identifies several variations of note. Compared to the overall average control group response rate of 14.97%, the median and range of control group response associated with some interventions (e.g. etanercept + methotrexate (MTX), etanercept monotherapy, MTX + sulfasalazine (SSZ) + hydroxychloroquine (HCQ), SSZ + HCQ, tocilizumab (TOC) 4 mg) was notably higher, while in other cases (e.g. certolizumab (CERTO) + MTX, golimumab+MTX, rituximab (RIT), RIT + MTX, TOC 8 mg, tofacitinib (TOF) + MTX) was notably lower. Estimated odds ratios summarizing eTach intervention’s relative treatment effect for ACR 50 response versus placebo are presented in Fig. 3, and demonstrate a strong inverse negative linear relationship between control group response rate and treatment effect. This finding provides strong support for the incorporation of an interaction term using meta-regression analysis that may prove of considerable value for evidence synthesis and decision-making. Additional file 1: Appendix 2 provides a summary of model fit information from both the unadjusted and adjusted NMA models.

### Findings from unadjusted NMA of ACR 50 response

**Fig. 4:**
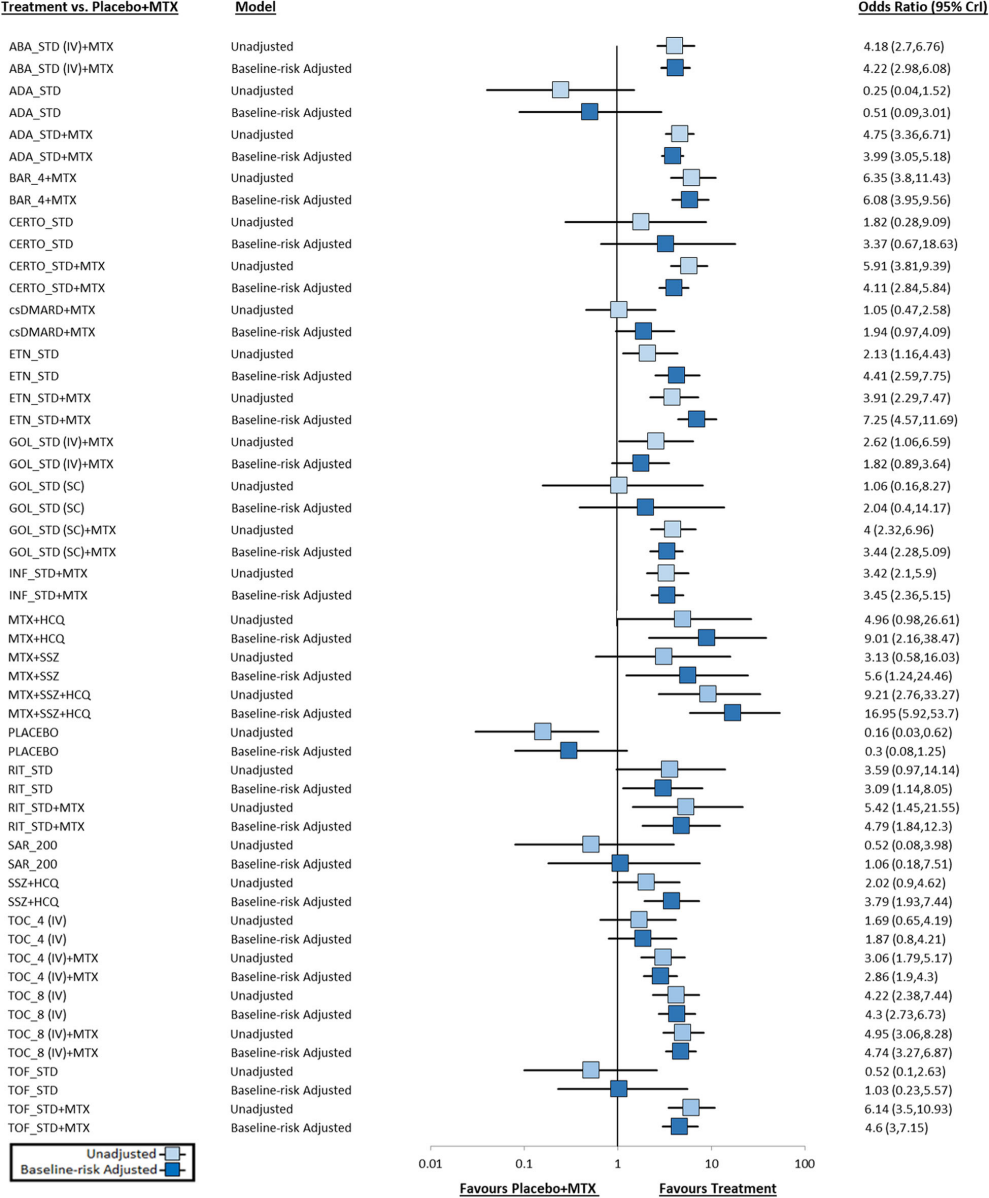
Forest Plot of ACR50 Treatment Effect Estimates (OR and 95% CrI) from Unadjusted and Adjusted NMAs. Odds ratios estimated from the unadjusted and control group risk adjusted RE NMAs are presented above, focused on comparisons of active interventions versus placebo. Values are reported with corresponding 95% credible intervals. Odds ratios and > 1 favor the active comparator. Downward and upward shifts in treatment effects between NMA approaches can be seen. Legend: ABA = abatacept; ADA = adalimumab; BAR_4 = 4 mg baricitinib; CERTO = certolizumab pegol; csDMARD = conventional synthetic disease-modifying anti-rheumatic drug; ETN = etanercept; GOL = golimumab; INF = infliximab; IV = intravenous; MTX = methotrexate; RIT = rituximab; SAR_200 = 200 mg sarilumab; SC = subcutaneous; SSZ = sulfasalazine; STD = standard dose; TOC_4 = tocilizumab 4 mg/kg; TOC_8 = 8 mg/kg tocilizumab; TOF = tofacitinib

**Fig. 5:**
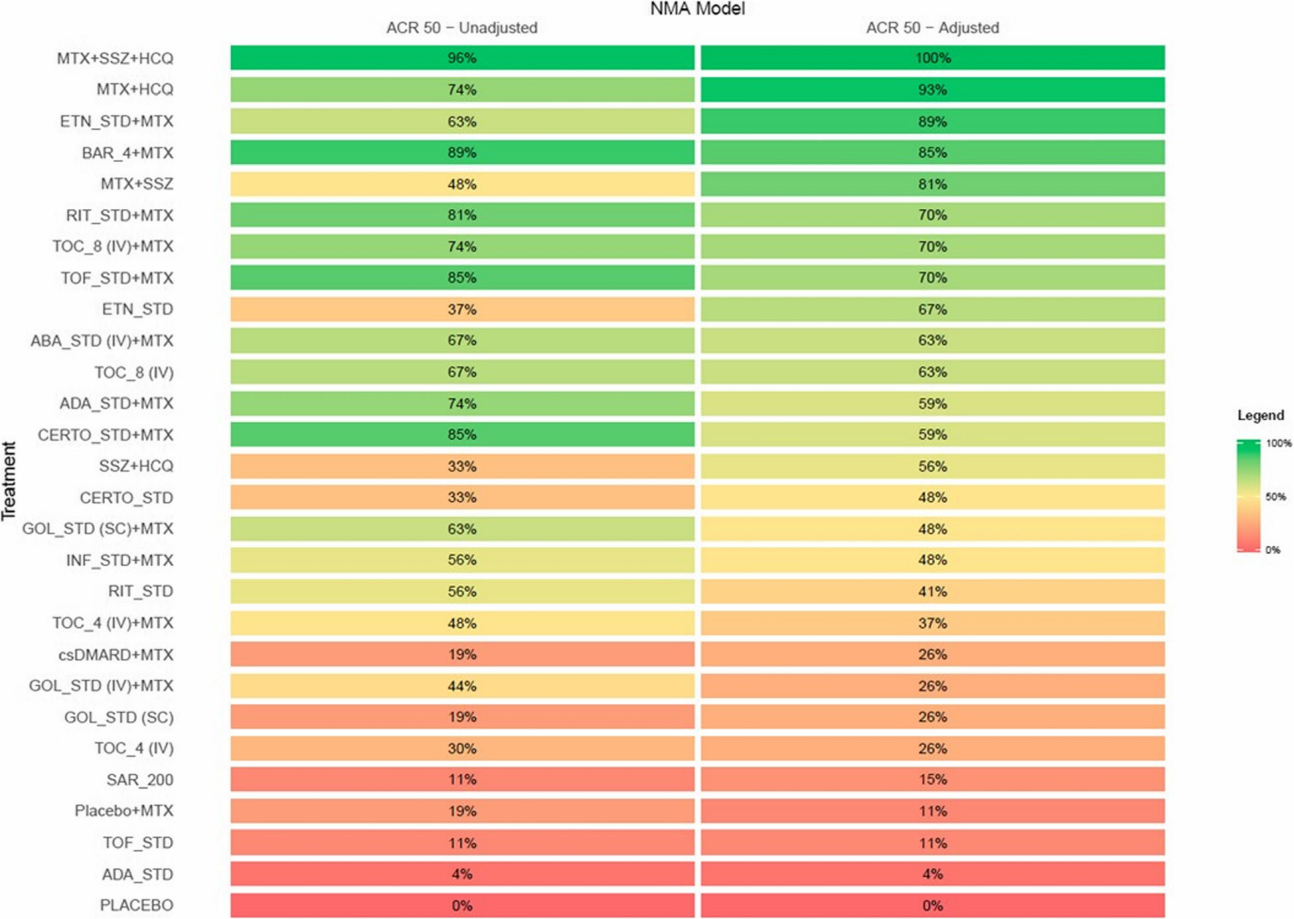
Heat Map of SUCRA Values per Intervention from Unadjusted and Adjusted NMAs, ACR 50 Response. SUCRA values from both unadjusted and baseline risk adjusted NMAs are presented using a heat map to allow visualization of treatment hierarchy as well as changes in SUCRA valus between models. Legend: ABA = abatacept; ADA = adalimumab; BAR_4 = 4 mg baricitinib; CERTO = certolizumab pegol; csDMARD = conventional synthetic disease-modifying anti-rheumatic drug; ETN = etanercept; GOL = golimumab; INF = infliximab; IV = intravenous; MTX = methotrexate; RIT = rituximab; SAR_200 = 200 mg sarilumab; SC = subcutaneous; SSZ = sulfasalazine; STD = standard dose; TOC_4 = tocilizumab 4 mg/kg; TOC_8 = 8 mg/kg tocilizumab; TOF = tofacitinib

The forest plot presented in Fig. 4 includes a summary of comparisons between biologics and placebo from the unadjusted (and adjusted) analysis using MTX + placebo as the reference group; Additional file 1: Appendix 3 presents a league table of all pairwise comparisons between biologics in the network. Nearly all active interventions were found to be associated with a greater likelihood of ACR 50 response compared to placebo, with ORs ranging in magnitude between 1.62 (95% CrI 0.50 to 5.00; adalimumab) and 59.81 (95% CrI 10.40 to 351.74; MTX + SSZ + HCQ). SUCRA values (see left panel of Fig. 5) ranged between 0 and 96%, with the highest ranked five interventions being MTX SSZ + HCQ (96%), baricitinib 4 mg + MTX (89%), TOF + MTX (85%), CERTO + MTX (85%), and RIT + MTX (81%); the five lowest-ranked interventions were placebo (0%), adalimumab (4%), TOF (11%), sarilumab 200 mg (11%) and placebo + MTX (19%).

### Findings from adjusted NMAs accounting for differences in baseline risk

To account for cross-study differences in control group response rate that were identified in Figs. 2 and 3, a meta-regression adjustment was introduced into the NMA RE model using an established extension of the unadjusted model used earlier.

The NMA incorporating adjustment for control group response rate was more reliable for decision-making purposes. The between-study standard deviation was reduced from 0.35 (95% CrI 0.19 to 0.55) to 0.29 (95% CrI 0.16 to 0.44), and the regression coefficient demonstrated a potentially important effect on model results (− 0.68, 95% CrI − 0.89 to − 0.44). A beta coefficient of − 0.68 (95% CrI − 0.89 to − 0.44) suggests that control group response is an important treatment effect modifier in NMAs of ACR50 response in rheumatoid arthritis and that lower control group response rates are associated with more favorable ORs than higher control group response rates (Additional file 1: Appendix 4).

In addition to results from the unadjusted NMA, the forest plot in Fig. 4 also presents a summary of the odds ratios for each intervention compared with placebo + MTX from the adjusted analysis, while corresponding SUCRA values are provided in the right panel of Fig. 5 (Additional file 1: Appendix 3 also provides a league table summary of all pairwise comparisons). Overall, an average change of 38.1% in the magnitude of the ORs between treatment comparisons (Additional file 1: Appendix 5). Notable gains can be seen for several treatments including etanercept + MTX (from 63 to 89%), MTX + HCQ (from 74 to 93%), MTX + SSZ (from 48 to 81%), etanercept monotherapy (from 37 to 67%) and SSZ + HCQ (from 33 to 56%), all of which were associated with increased or comparable ranges of control group response rate relative to the overall average rate across trials; conversely, sizable reductions were observed for RIT + MTX, TOF + MTX, ADA + MTX and CERTO + MTX, amongst other interventions. The five most highly ranked interventions based upon SUCRA value were MTX + SSZ + HCQ (100%), MTX + HCQ (93%), etanercept + MTX (89%), baricitinib 4 mg (85%) and MTX + SSZ (81%).

Several changes in clinical interpretations drawn from the unadjusted model were noted with regard to estimated treatment effects. There has been significant variability in the placebo response rates of RA clinical trials. For example, the odds ratio’s for adalimumab plus methotrexate versus etanercept plus methotrexate changes from 1.21 (95% CrI 0.60 to 2.23) favoring adalimumab in unadjusted NMA to 1.82 (95% CrI 1.06 to 3.14) favoring etanercept after adjustment in NMA.

Additional file 1 Appendix 6 provides an analogous description of findings from unadjusted and adjusted NMAs conducted with the same network of therapies, with the incorporation of biosimilars. Similar improvements in model fit and shifting of point estimates and SUCRA values were observed.

## Discussion

In the current study, we re-created an NMA comparing biologic interventions for moderate-to-severe RA [13]. In addition to re-creating this analysis, the data from this study were used to demonstrate approaches to inspecting for the presence of cross-study variability in control group risk, as well as the importance of accounting for its presence in the context of NMAs in general and with respect to RA. This study will add to past research that has discussed the importance of addressing cross-trial heterogeneity in NMA [6, 8, 13].

As NMAs continue to become increasingly common regarding their use to compare healthcare interventions and more researchers develop an interest in their implementation, there is a need to encourage rigorous efforts for modeling when cross-study variability exists. If researchers undertaking NMAs fail to inspect data sets for such variability carefully, then the risk of presenting and drawing interpretations from potentially misleading estimates of treatment effect from NMAs increases. In presenting the current case study, we hope to add to past literature that has noted the value and importance of exploring covariate adjustments in NMA in general and to re-emphasize their importance in the context of analyses seeking to compare biologic interventions for RA.

The current case study of biologics for RA presents an illustration of an NMA wherein considerable cross-study heterogeneity was identified regarding ACR50 control group response. Network structure did not allow us to adjust for multiple characteristics simultaneously but allowed for adjustment of control group response which serves as a proxy for differences in multiple characteristics. We focused on ACR 50 because that was the primary outcome in the CADTH therapeutic review [12]. In the original review, adjustments for control group response rate were not performed [12]. The NICE TSD series have previously identified analyses of ACR outcomes in RA as a scenario wherein analyses accounting for this source of variability should be considered as the primary analysis from which interpretations should be drawn [6]. Other guidance documents have also addressed the importance of accounting for the presence of heterogeneity [14–16]. It is very evident from box plots (Fig. 2) and scatterplots of effect estimates about ACR 50 control group response across trials (Fig. 3) that control group response rate is related to treatment effect. Not surprisingly, a meta-regression adjusting to account for this relationship was associated with an improved model fit (associated with statistically significant regression coefficient and a reduction in the between-study variance parameter). Lack of adjustment for cross-trial differences was associated with different clinical interpretations of findings from NMA, demonstrating a bias against interventions which reported higher ACR 50 response rates in the control group (e.g., etanercept).

The findings of this study have important implications for HTA agencies where NMAs are often incorporated into health economic evaluations. As noted in NICE TSDs, cost-effectiveness estimates from an unadjusted NMA will be very different compared to an NMA adjusting for differences in patient characteristics across studies. For example, in our case study, the relative risk of adalimumab plus methotrexate versus etanercept plus methotrexate changes from favoring adalimumab in unadjusted NMA to favoring etanercept after adjustment in NMA. Given health economic evaluations are driven by mean treatment effects, the naïve use of an unadjusted NMA in a cost-effectiveness analysis could fundamentally result in an author incorrectly concluding that etanercept is more expensive and less effective than adalimumab. It is therefore imperative that authors of cost-effectiveness analyses in RA assess whether NMAs have adequately adjusted for differences in patient populations before using to populate their economic models. It is reassuring that the importance of adjustment for control group risk has been recognized by NICE [6]; they indicate that investigations of interventions for RA should clearly identify a relationship between the efficacy of interventions and control group risk “that needs to be incorporated into cost-effectiveness analyses”.

As others have recommended previously, the current case study provides strong support that NMAs of ACR outcomes in the realm of moderate-to-severe RA should be based upon a model accounting for cross-study differences in baseline risk, and when uncertain of this, that authors should undertake inspections of heterogeneity between studies to assess its presence. Variability in patients baseline demographics are known in general to have the ability to impact findings within both clinical trials and knowledge syntheses, and in the context of NMA, adjustments for control group risk can function as a proxy measure, capturing the effects of several relevant known (e.g., duration of rheumatoid arthritis, biologic experience) and unknown factors simultaneously. This is advantageous because it permits adjustment for multiple clinical characteristics which are relevant in RA that is not possible to adjust for using meta-regressions on individual characteristics due to network structure (i.e., often only enough studies to adjust in meta-regression for one variable). There was strong support observed in Fig. 3 for the incorporation of an interaction term in NMA using meta-regression, and changes in estimates of treatment effect observed in Fig. 4 clearly show that this approach has important implications for clinical decision-making and economic evaluation of biologic interventions for RA. Therefore, adjustment for baseline risk likely represents an especially important adjustment factor in scenarios wherein assorted cross-trial differences between study populations are known to be present.

In general, adjusting for baseline-risk in meta-regression is most useful when: a) networks include one or more connections with many studies (e.g., greater than 5); b) there is spread across studies in terms of control group response rate, and; c) there is a relationship between control group response rate and treatment effect. Fortunately, the ACR50 network in rheumatoid arthritis meets all these criteria and it is worthwhile to conduct in the example here, but that may not always be the case in other therapeutic areas. Indeed, in many therapeutic areas, there won’t be a sufficient number of studies in the network to conduct a meta-regression adjusting for baseline-risk, and methods leveraging individual patient data will be required to adequately adjust for heterogeneity [17].

## Conclusions

When comparing findings from a collection of RCTs which consist of heterogeneous patient populations, the use of an NMA that does not properly adjust for patient characteristics is likely to produce estimates of treatment effect that may be biased. Efforts should be taken to account and adjust for sources of heterogeneity, especially when they are well established and accepted in the literature. Use of an NMA model accounting for cross-study variability in control group response, which can account for both observed and unobserved confounders, was associated with important gains in model fit as well as several significant shifts in clinical interpretation. Future clinical systematic reviews and health technology assessments related to the comparison of interventions for rheumatoid arthritis should consider adjustment for control group response rate when conducting NMAs.

## Supplementary information

**Additional file 1: Appendix 1.** List of Studies Included in the NMA. **Appendix 2.** Summary of Model Fit Information from Unadjusted and Adjusted NMA Models. **Appendix 3.** League Table Summary of Findings from NMA Accounting for Differences in Control Group Response Rate. **Appendix 4.** Coefficient Interpretation - Baseline Response vs. Treatment Effect Plot. **Appendix 5.** ACR 50 – OR CrI Median Percent Change League Table. **Appendix 6.** Supporting Figures and Findings for Sensitivity Analysis Including Biosimilar Agents.

## Data Availability

All data generated or analyzed during this study were based on publicly available summary data reported in publications. Data available on request.

## Abbreviations

ABA: Abatacept
ABP501: AnBaiNuo (biosimilar adalimumab)
ACR: American College of Rheumatology
ADA: Adalimumab
BAR_4: 4 mg baracitinib
CADTH: Canadian Agency for Drugs and Technologies in Health
CERTO: Certolizumab pegol
CrI: Credible interval
csDMARD: Conventional synthetic disease-modifying antirheumatic drug
CT-P13: Infliximab biosimilar
DMARD: Disease-modifying antirheumatic drug
DSU: Decision Support Unit
ETN: Etanercept
GOL: Golimumab
HCQ: Hydroxychloroquine
HD203: Etanercept biosimilar
HTA: Health technology assessment
ICER: Institute for Clinical and Economic Review
INF: Infliximab
IV: Intravenous
MCMC: Markov Chain Monte Carlo
mg: Milligram
MTX: Methotrexate
NICE: National Institute for Health and Care Excellence
NMA: Network meta-analysis
OR: Odds ratio
RA: Rheumatoid arthritis
RCT: Randomized controlled trial
RIT: Rituximab
SAR_200: 200 mg sarilumab
SB2: Biosimilar infliximab 3 mg/kg
SB4: Biosimilar etanercept 50 mg
SB5: Biosimilar adalimumab
SC: Subcutaneous
SEC: Secukinumab
SLR: Systematic literature review
SSZ: Sulfasalazine
STD: Standard dose
SUCRA: Surface Under the Cumulative Ranking
TOC: Tocilizumab
TOC_4: 4 mg/kg tocilizumab
TOC_8: 8 mg/kg tocilizumab
TOF: Tofacitinib
TSD: Technical Support Document
UST: Ustekinumab
ZRC-3197: Biosimilar of adalimumab
