# Supplementary material for: Incorporating adjustments for variability in control group response rates in network meta-analysis: a case study of biologics for rheumatoid arthritis
Source: BMC Med Res Methodol. 2019 Oct 16;19:193. doi: 10.1186/s12874-019-0837-2 (PMC6796442; doi:10.1186/s12874-019-0837-2)
Supplement: Supplementary file 1 — Additional file 1: Appendix 1. List of Studies Included in the NMA. Appendix 2. Summary of Model Fit Information from Unadjusted and Adjusted NMA Models. Appendix 3. League Table Summary of Findings from NMA Accounting for Differences in Control Group Response Rate. Appendix 4. Coefficient Interpretation - Baseline Response vs. Treatment Effect Plot. Appendix 5. ACR 50 – OR CrI Median Percent Change League Table. Appendix 6. Supporting Figures and Findings for Sensitivity Analysis Including Biosimilar Agents. [file 12874_2019_837_MOESM1_ESM.docx]

**Additional file 1**

- **Appendix 1**: List of Studies Included in the NMA
- **Appendix 2**: Summary of Model Fit Information from Unadjusted and Adjusted NMA Models
- **Appendix 3**: League Table Summary of Findings from NMA Accounting for Differences in Control Group Response Rate
- **Appendix 4:** Coefficient Interpretation - Baseline Response vs. Treatment Effect Plot
- **Appendix 5**: ACR 50 – OR CrI Median Percent Change League Table
- **Appendix 6**: Supporting Figures and Findings for Sensitivity Analysis Including Biosimilar Agents

**Appendix 1: List of Studies Included in the NMA**

1. Abe T, Takeuchi T, Miyasaka N, Hashimoto H, Kondo H, Ichikawa Y, et al. A multicenter, double-blind, randomized, placebo controlled trial of infliximab combined with low dose methotrexate in japanese patients with rheumatoid arthritis. J Rheumatol 2006;33:37-44.
2. Burmester GR, Lin Y, Patel R, van Adelsberg J, Mangan EK, Graham NM, et al. Efficacy and safety of sarilumab monotherapy versus adalimumab monotherapy for the treatment of patients with active rheumatoid arthritis (monarch): A randomised, double-blind, parallel-group phase iii trial. Ann Rheum Dis 2017;76:840-7.
3. Chen DY, Chou SJ, Hsieh TY, Chen YH, Chen HH, Hsieh CW, et al. Randomized, double-blind, placebo-controlled, comparative study of human anti-tnf antibody adalimumab in combination with methotrexate and methotrexate alone in taiwanese patients with active rheumatoid arthritis. J Formos Med Assoc 2009;108:310-9.
4. VanLunen B, Choy E, McKenna F, Stahl H-D, Vencovsky J, Goel N, et al. Certolizumab pegol plus mtx administered every 4 weeks is effective in patients with ra who are partial responders to mtx. Rheumatology 2012;51:1226-34.
5. Dougados M, Kissel K, Sheeran T, Tak PP, Conaghan PG, Mola EM, et al. Adding tocilizumab or switching to tocilizumab monotherapy in methotrexate inadequate responders: 24-week symptomatic and structural results of a 2-year randomised controlled strategy trial in rheumatoid arthritis (act-ray). Ann Rheum Dis 2013;72:43-50.
6. Fleischmann R, Vencovsky J, van Vollenhoven RF, Borenstein D, Box J, Coteur G, et al. Efficacy and safety of certolizumab pegol monotherapy every 4 weeks in patients with rheumatoid arthritis failing previous disease-modifying antirheumatic therapy: The fast4ward study. Ann Rheum Dis 2009;68:805-11.
7. Fleischmann R, Cutolo M, Genovese MC, Lee EB, Kanik KS, Sadis S, et al. Phase iib dose-ranging study of the oral jak inhibitor tofacitinib (cp-690,550) or adalimumab monotherapy versus placebo in patients with active rheumatoid arthritis with an inadequate response to disease-modifying antirheumatic drugs. Arthritis Rheum 2012;64:617-29.
8. Ueki Y, Saito K, Nagaoka S, Hidaka T, Atsumi T, Tsukano M, et al. Etanercept (etn) with methotrexate (mtx) is better than etn monotherapy in patients with active rheumatoid arthritis despite mtx therapy: A randomized trial au - kameda, hideto. Modern Rheumatology 2010;20:531-8.
9. Kaneko Y, Atsumi T, Tanaka Y, Inoo M, Kobayashi-Haraoka H, Amano K, et al. Comparison of adding tocilizumab to methotrexate with switching to tocilizumab in patients with rheumatoid arthritis with inadequate response to methotrexate: 52-week results from a prospective, randomised, controlled study (surprise study). Ann Rheum Dis 2016;75:1917-23.
10. Kay J, Matteson EL, Dasgupta B, Nash P, Durez P, Hall S, et al. Golimumab in patients with active rheumatoid arthritis despite treatment with methotrexate: A randomized, double-blind, placebo-controlled, dose-ranging study. Arthritis Rheum 2008;58:964-75.
11. Keystone EC, Genovese MC, Klareskog L, Hsia EC, Hall ST, Miranda PC, et al. Golimumab, a human antibody to tumour necrosis factor alpha given by monthly subcutaneous injections, in active rheumatoid arthritis despite methotrexate therapy: The go-forward study. Ann Rheum Dis 2009;68:789-96.
12. Keystone EC, Taylor PC, Drescher E, Schlichting DE, Beattie SD, Berclaz P-Y, et al. Safety and efficacy of baricitinib at 24 weeks in patients with rheumatoid arthritis who have had an inadequate response to methotrexate. Annals of the rheumatic diseases 2015;74:333-40.
13. Keystone EC, Kavanaugh AF, Sharp JT, Tannenbaum H, Hua Y, Teoh LS, et al. Radiographic, clinical, and functional outcomes of treatment with adalimumab (a human anti-tumor necrosis factor monoclonal antibody) in patients with active rheumatoid arthritis receiving concomitant methotrexate therapy: A randomized, placebo-controlled, 52-week trial. Arthritis Rheum 2004;50:1400-11.
14. Keystone E, Heijde DVD, Mason Jr D, Landewé R, Vollenhoven RV, Combe B, et al. Certolizumab pegol plus methotrexate is significantly more effective than placebo plus methotrexate in active rheumatoid arthritis: Findings of a fifty-two–week, phase iii, multicenter, randomized, double-blind, placebo-controlled, parallel-group study. Arthritis & Rheumatism 2008;58:3319-29.
15. Kim HY, Hsu PN, Barba M, Sulaiman W, Robertson D, Vlahos B, et al. Randomized comparison of etanercept with usual therapy in an asian population with active rheumatoid arthritis: The appeal trial. Int J Rheum Dis 2012;15:188-96.
16. Klareskog L, van der Heijde D, de Jager JP, Gough A, Kalden J, Malaise M, et al. Therapeutic effect of the combination of etanercept and methotrexate compared with each treatment alone in patients with rheumatoid arthritis: Double-blind randomised controlled trial. Lancet (London, England) 2004;363:675-81.
17. Kremer JM, Blanco R, Brzosko M, Burgos-Vargas R, Halland AM, Vernon E, et al. Tocilizumab inhibits structural joint damage in rheumatoid arthritis patients with inadequate responses to methotrexate: Results from the double-blind treatment phase of a randomized placebo-controlled trial of tocilizumab safety and prevention of structural joint damage at one year. Arthritis Rheum 2011;63:609-21.
18. Kremer JM, Westhovens R, Leon M, Di Giorgio E, Alten R, Steinfeld S, et al. Treatment of rheumatoid arthritis by selective inhibition of t-cell activation with fusion protein ctla4ig. N Engl J Med 2003;349:1907-15.
19. Kremer JM, Genant HK, Moreland LW, Russell AS, Emery P, Abud-Mendoza C, et al. Effects of abatacept in patients with methotrexate-resistant active rheumatoid arthritis: A randomized trial. Ann Intern Med 2006;144:865-76.
20. Lan JL, Chou SJ, Chen DY, Chen YH, Hsieh TY, Young M, Jr. A comparative study of etanercept plus methotrexate and methotrexate alone in taiwanese patients with active rheumatoid arthritis: A 12-week, double-blind, randomized, placebo-controlled study. J Formos Med Assoc 2004;103:618-23.
21. Li Z, Zhang F, Kay J, Fei K, Han C, Zhuang Y, et al. Efficacy and safety results from a phase 3, randomized, placebo-controlled trial of subcutaneous golimumab in chinese patients with active rheumatoid arthritis despite methotrexate therapy. Int J Rheum Dis 2016;19:1143-56.
22. Machado DA, Guzman RM, Xavier RM, Simon JA, Mele L, Pedersen R, et al. Open-label observation of addition of etanercept versus a conventional disease-modifying antirheumatic drug in subjects with active rheumatoid arthritis despite methotrexate therapy in the latin american region. J Clin Rheumatol 2014;20:25-33.
23. Maini R, St Clair EW, Breedveld F, Furst D, Kalden J, Weisman M, et al. Infliximab (chimeric anti-tumour necrosis factor alpha monoclonal antibody) versus placebo in rheumatoid arthritis patients receiving concomitant methotrexate: A randomised phase iii trial. Attract study group. Lancet (London, England) 1999;354:1932-9.
24. Maini RN, Taylor PC, Szechinski J, Pavelka K, Bröll J, Balint G, et al. Double-blind randomized controlled clinical trial of the interleukin-6 receptor antagonist, tocilizumab, in european patients with rheumatoid arthritis who had an incomplete response to methotrexate. Arthritis & Rheumatism 2006;54:2817-29.
25. Moreland LW, Schiff MH, Baumgartner SW, Tindall EA, Fleischmann RM, Bulpitt KJ, et al. Etanercept therapy in rheumatoid arthritis. A randomized, controlled trial. Ann Intern Med 1999;130:478-86.
26. Nishimoto N, Miyasaka N, Yamamoto K, Kawai S, Takeuchi T, Azuma J, et al. Study of active controlled tocilizumab monotherapy for rheumatoid arthritis patients with an inadequate response to methotrexate (satori): Significant reduction in disease activity and serum vascular endothelial growth factor by il-6 receptor inhibition therapy. Mod Rheumatol 2009;19:12-9.
27. Schiff M, Keiserman M, Codding C, Songcharoen S, Berman A, Nayiager S, et al. Efficacy and safety of abatacept or infliximab vs placebo in attest: A phase iii, multi-centre, randomised, double-blind, placebo-controlled study in patients with rheumatoid arthritis and an inadequate response to methotrexate. Ann Rheum Dis 2008;67:1096-103.
28. Smolen J, Landewe RB, Mease P, Brzezicki J, Mason D, Luijtens K, et al. Efficacy and safety of certolizumab pegol plus methotrexate in active rheumatoid arthritis: The rapid 2 study. A randomised controlled trial. Ann Rheum Dis 2009;68:797-804.
29. Smolen JS, Beaulieu A, Rubbert-Roth A, Ramos-Remus C, Rovensky J, Alecock E, et al. Effect of interleukin-6 receptor inhibition with tocilizumab in patients with rheumatoid arthritis (option study): A double-blind, placebo-controlled, randomised trial. Lancet (London, England) 2008;371:987-97.
30. Smolen JS, Burmester GR, Combe B, Curtis JR, Hall S, Haraoui B, et al. Head-to-head comparison of certolizumab pegol versus adalimumab in rheumatoid arthritis: 2-year efficacy and safety results from the randomised exxelerate study. Lancet (London, England) 2016;388:2763-74.
31. Strand V, Balbir-Gurman A, Pavelka K, Emery P, Li N, Yin M, et al. Sustained benefit in rheumatoid arthritis following one course of rituximab: Improvements in physical function over 2 years. Rheumatology (Oxford) 2006;45:1505-13.
32. Takeuchi T, Harigai M, Tanaka Y, Yamanaka H, Ishiguro N, Yamamoto K, et al. Golimumab monotherapy in japanese patients with active rheumatoid arthritis despite prior treatment with disease-modifying antirheumatic drugs: Results of the phase 2/3, multicentre, randomised, double-blind, placebo-controlled go-mono study through 24 weeks. Annals of the rheumatic diseases 2013;72:1488-95.
33. Takeuchi T, Matsubara T, Nitobe T, Suematsu E, Ohta S, Honjo S, et al. Phase ii dose-response study of abatacept in japanese patients with active rheumatoid arthritis with an inadequate response to methotrexate. Mod Rheumatol 2013;23:226-35.
34. Tanaka Y, Suzuki M, Nakamura H, Toyoizumi S, Zwillich SH, Tofacitinib Study I. Phase ii study of tofacitinib (cp-690,550) combined with methotrexate in patients with rheumatoid arthritis and an inadequate response to methotrexate. Arthritis Care Res (Hoboken) 2011;63:1150-8.
35. Tanaka Y, Harigai M, Takeuchi T, Yamanaka H, Ishiguro N, Yamamoto K, et al. Golimumab in combination with methotrexate in japanese patients with active rheumatoid arthritis: Results of the go-forth study. Ann Rheum Dis 2012;71:817-24.
36. Taylor PC, Keystone EC, van der Heijde D, Weinblatt ME, Del Carmen Morales L, Reyes Gonzaga J, et al. Baricitinib versus placebo or adalimumab in rheumatoid arthritis. N Engl J Med 2017;376:652-62.
37. van Riel PL, Taggart AJ, Sany J, Gaubitz M, Nab HW, Pedersen R, et al. Efficacy and safety of combination etanercept and methotrexate versus etanercept alone in patients with rheumatoid arthritis with an inadequate response to methotrexate: The adore study. Ann Rheum Dis 2006;65:1478-83.
38. van Vollenhoven RF, Kinnman N, Vincent E, Wax S, Bathon J. Atacicept in patients with rheumatoid arthritis and an inadequate response to methotrexate: Results of a phase ii, randomized, placebo-controlled trial. Arthritis Rheum 2011;63:1782-92.
39. van Vollenhoven RF, Fleischmann R, Cohen S, Lee EB, Garcia Meijide JA, Wagner S, et al. Tofacitinib or adalimumab versus placebo in rheumatoid arthritis. N Engl J Med 2012;367:508-19.
40. Weinblatt ME, Kremer JM, Bankhurst AD, Bulpitt KJ, Fleischmann RM, Fox RI, et al. A trial of etanercept, a recombinant tumor necrosis factor receptor:Fc fusion protein, in patients with rheumatoid arthritis receiving methotrexate. N Engl J Med 1999;340:253-9.
41. Weinblatt ME, Bingham CO, 3rd, Mendelsohn AM, Kim L, Mack M, Lu J, et al. Intravenous golimumab is effective in patients with active rheumatoid arthritis despite methotrexate therapy with responses as early as week 2: Results of the phase 3, randomised, multicentre, double-blind, placebo-controlled go-further trial. Ann Rheum Dis 2013;72:381-9.
42. Weinblatt ME, Keystone EC, Furst DE, Moreland LW, Weisman MH, Birbara CA, et al. Adalimumab, a fully human anti-tumor necrosis factor alpha monoclonal antibody, for the treatment of rheumatoid arthritis in patients taking concomitant methotrexate: The armada trial. Arthritis Rheum 2003;48:35-45.
43. Weinblatt ME, Mease P, Mysler E, Takeuchi T, Drescher E, Berman A, et al. The efficacy and safety of subcutaneous clazakizumab in patients with moderate-to-severe rheumatoid arthritis and an inadequate response to methotrexate: Results from a multinational, phase iib, randomized, double-blind, placebo/active-controlled, dose-ranging study. Arthritis Rheumatol 2015;67:2591-600.
44. Takeuchi T, Yamanaka H, Ishiguro N, Tanaka Y, Eguchi K, Watanabe A, et al. Efficacy and safety of certolizumab pegol plus methotrexate in japanese rheumatoid arthritis patients with an inadequate response to methotrexate: The j-rapid randomized, placebo-controlled trial au - yamamoto, kazuhiko. Modern Rheumatology 2014;24:715-24.
45. Yamamoto K, Takeuchi T, Yamanaka H, Ishiguro N, Tanaka Y, Eguchi K, et al. Efficacy and safety of certolizumab pegol plus methotrexate in japanese rheumatoid arthritis patients with an inadequate response to methotrexate: The j-rapid randomized, placebo-controlled trial. Mod Rheumatol 2014;24:715-24.

**Appendix 2: Summary of Model Fit Information from Unadjusted and Adjusted NMA Models**

The table below provides a summary of the model fit information that was used to compare the fit of unadjusted and adjusted NMA models, as well as to evaluate the importance of a regression adjustment for control group response rate in terms of both its estimated magnitude and impact on the between-study standard deviation parameter.

| **Parameter** | **Estimates Corresponding to Unadjusted NMA** | **Estimates Corresponding to Baseline-risk Adjusted NMA** |
| --- | --- | --- |
| Beta (95% CrI) | NA | -0.68 (-0.89 to -0.44) |
| Heterogeneity (SD, 95% CrI) | 0.35 (0.19 to 0.55) | 0.29 (0.16 to 0.44) |
| Total residual deviance (versus 119 data points | 122.80 | 122.86 |
| DIC | 774.80 | 778.16 |

**Appendix 3: League Table Summary of Findings from NMA Accounting for Differences in Control Group Response Rate**

**Unadjusted NMA Results**

**
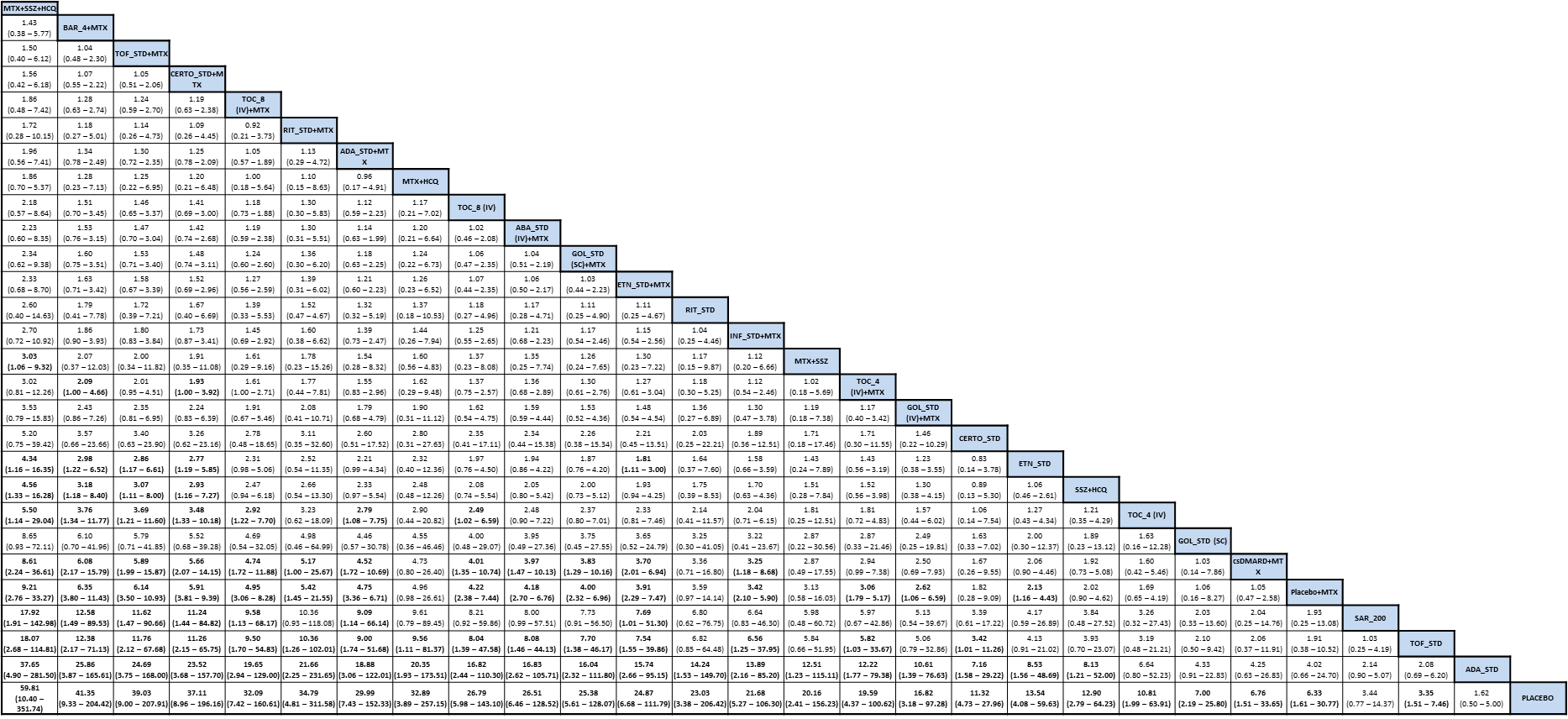
**

Odds ratios from network meta-analyses for rheumatoid arthritis for all pairwise comparisons. ORs for ACR50 are below the diagonal (row-defining treatment vs. column-defining treatment) and ORs> 1 means treatment in the top is better. To obtain ORs for comparisons in the opposite direction, reciprocals can be taken. Significant results are in bold.

**Legend:** ABA = abatacept; ADA = adalimumab; BAR_4 = 4mg baricitinib; CERTO = certolizumab pegol; csDMARD = conventional synthetic disease-modifying anti-rheumatic drug; ETN=etanercept; GOL = golimumab; INF = infliximab; IV = intravenous; MTX = methotrexate; RIT = rituximab; SAR_200 = 200mg sarilumab; SC = subcutaneous; SSZ = sulfasalazine; STD = standard dose; TOC_4 = tocilizumab 4mg/kg; TOC_8 = 8mg/kg tocilizumab; TOF = tofacitinib.

**Meta-Regression Adjusted NMA Results**

**
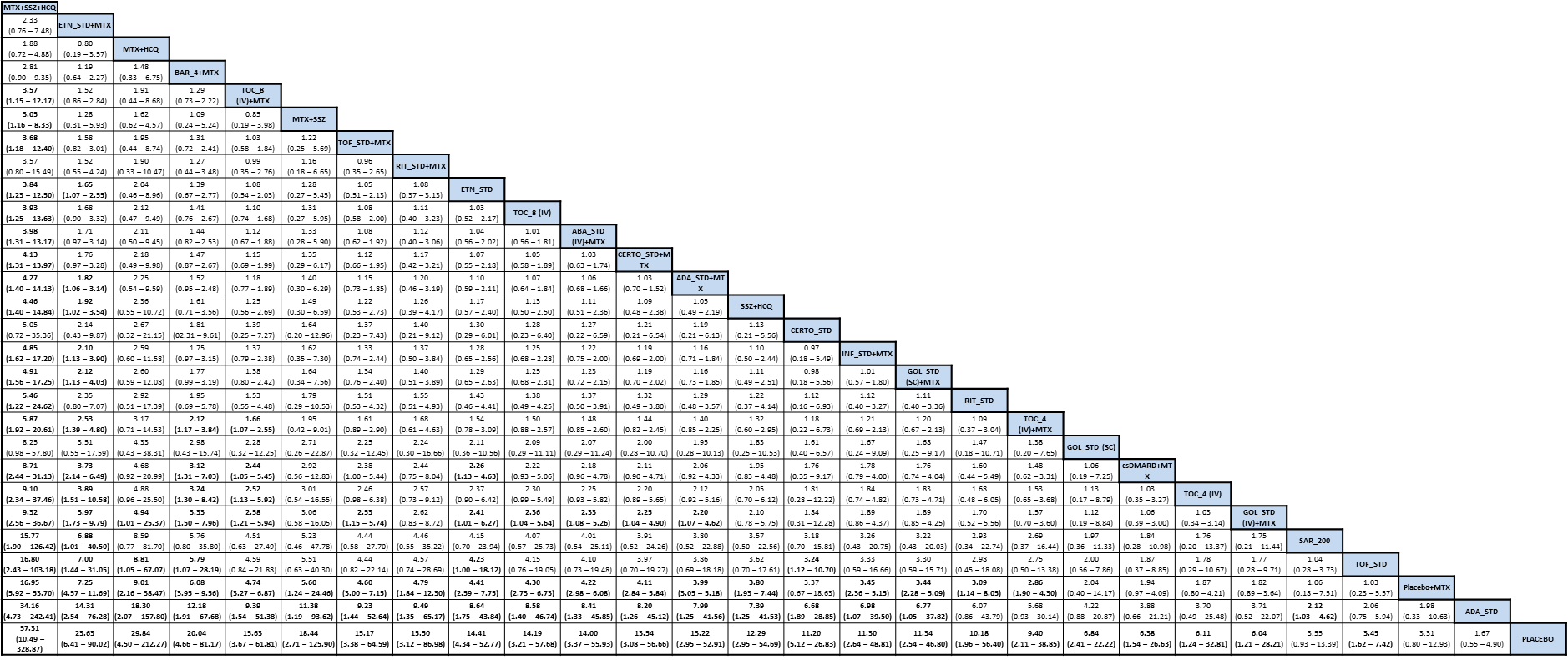
**

Odds ratios from network meta-analyses for rheumatoid arthritis for all pairwise comparisons. ORs for ACR50 are below the diagonal (row-defining treatment vs. column-defining treatment) and ORs> 1 means treatment in the top is better. To obtain ORs for comparisons in the opposite direction, reciprocals can be taken. Significant results are in bold.

**Legend:** ABA = abatacept; ADA = adalimumab; BAR_4 = 4mg baricitinib; CERTO = certolizumab pegol; csDMARD = conventional synthetic disease-modifying anti-rheumatic drug; ETN=etanercept; GOL = golimumab; INF = infliximab; IV = intravenous; MTX = methotrexate; RIT = rituximab; SAR_200 = 200mg sarilumab; SC = subcutaneous; SSZ = sulfasalazine; STD = standard dose; TOC_4 = tocilizumab 4mg/kg; TOC_8 = 8mg/kg tocilizumab; TOF = tofacitinib.

**Appendix 4: Coefficient Interpretation - Baseline Response vs. Treatment Effect Plot**

**
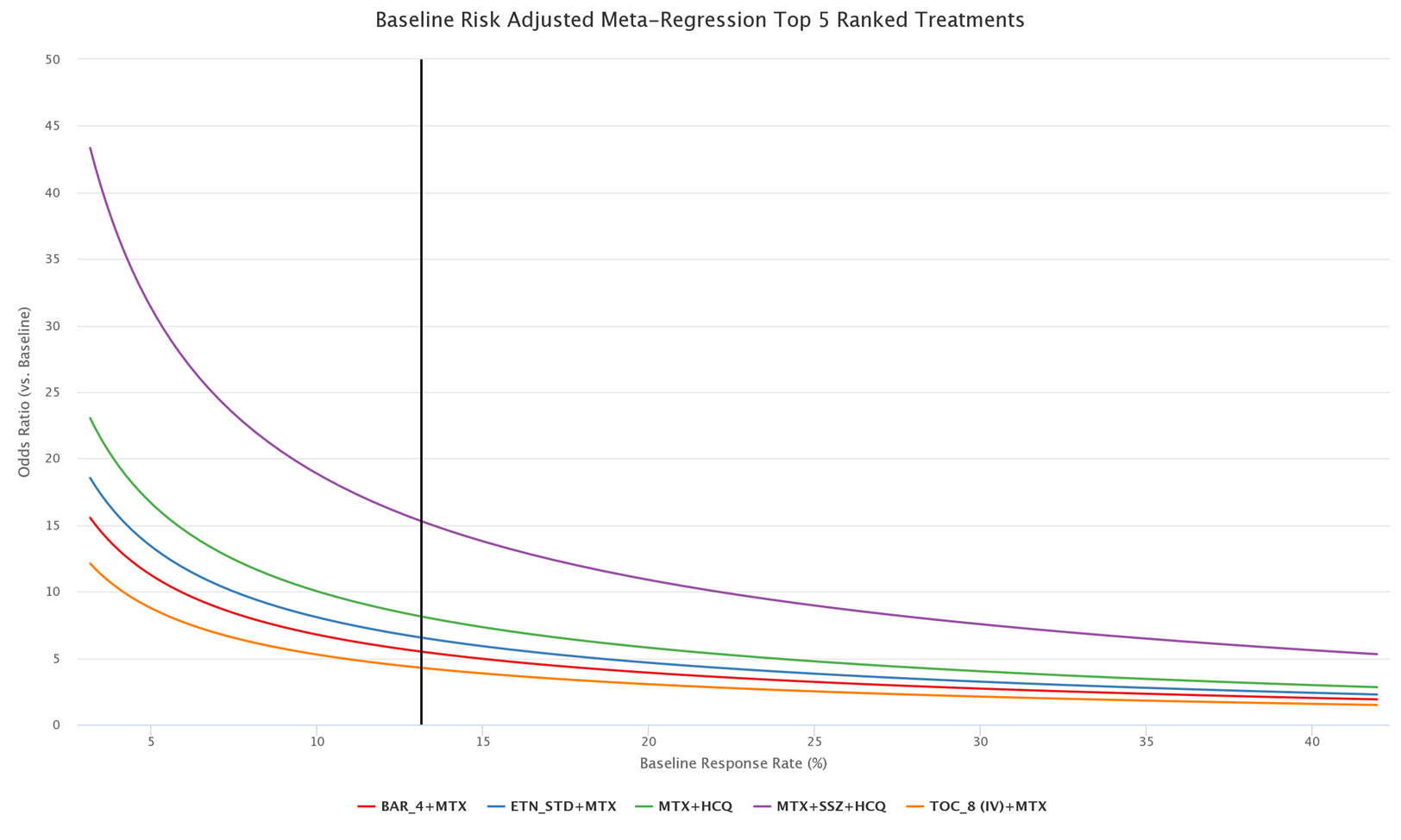
**

Average baseline response rate from data (13.15%)

**Appendix 5: ACR 50 – OR CrI Median Percent Change League Table**


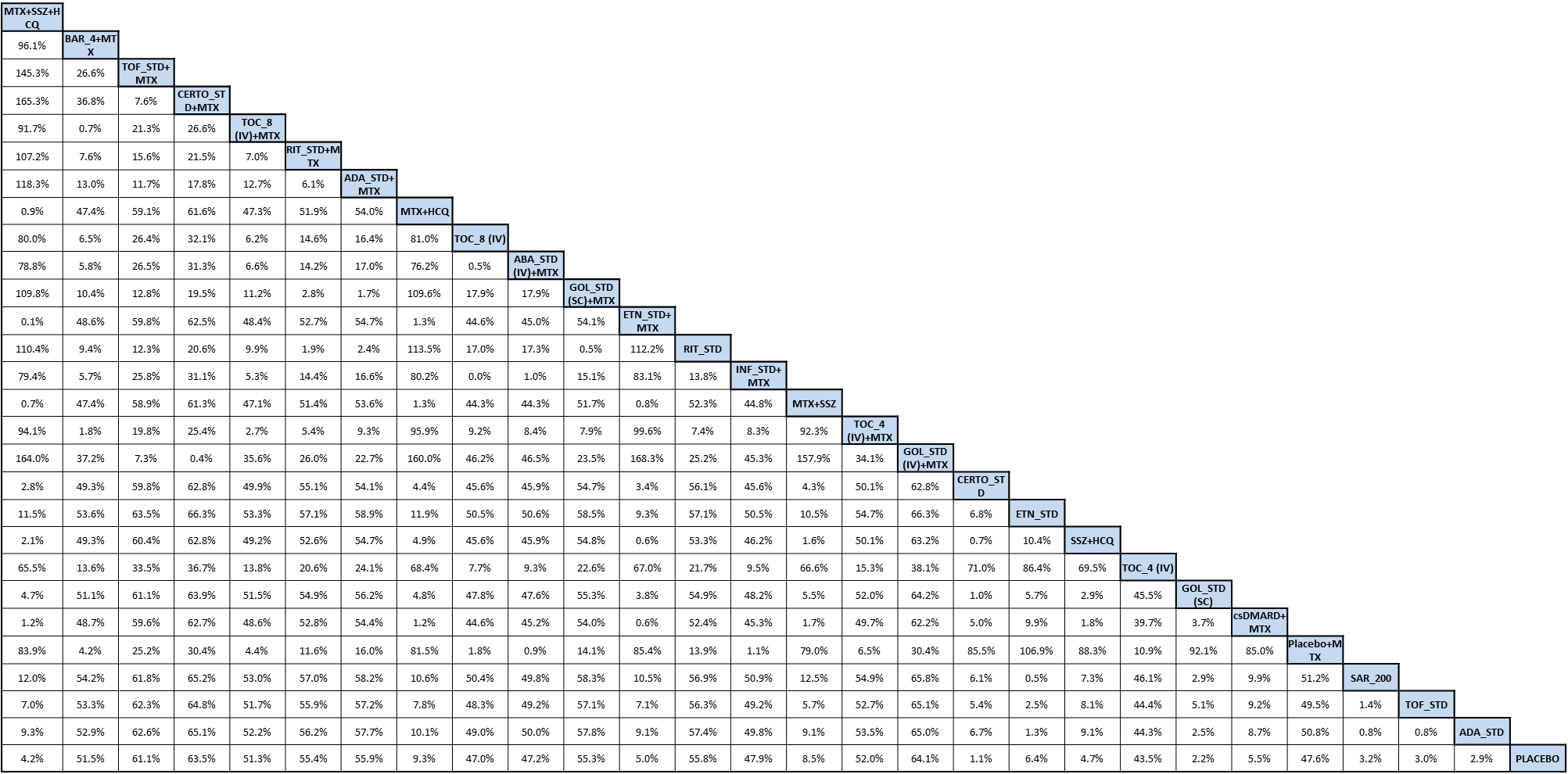


**Appendix 6:**

**Supporting Figures and Findings for Sensitivity Analysis Including Biosimilar Agents**

**Evidence Network Comparing Biologics for Moderate to Severe Rheumatoid Arthritis,**

**ACR50 Response (Including Biosimilars)**


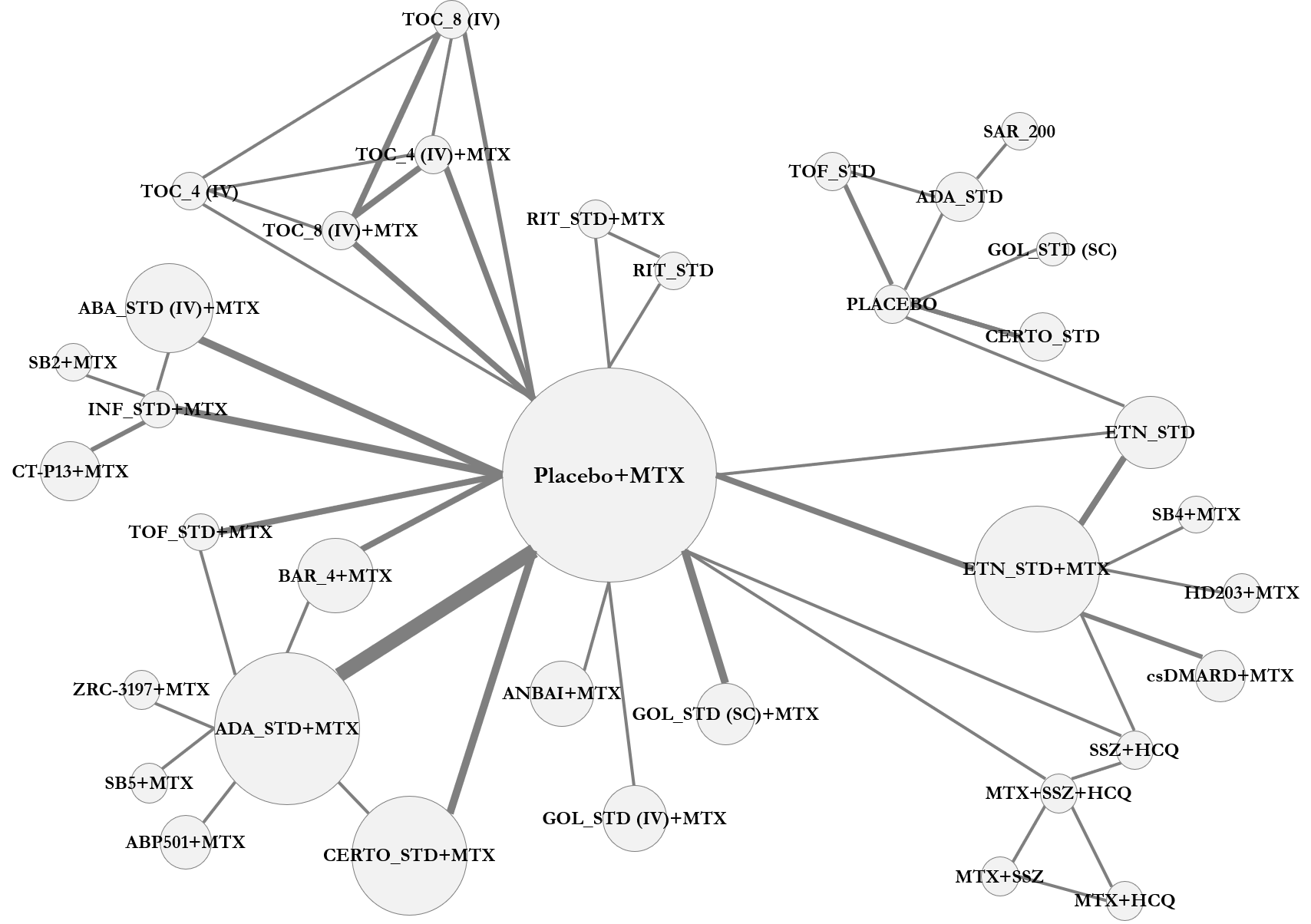


Treatment nodes are sized to proportionally reflect the numbers of patients randomized to each intervention, while the line thickness of edges joining nodes are asized to propotionally reflect the numbers of studies informing each comparison. Biosimilars were included in this sensitivity analysis.

**Legend**: ABA = abatacept; ABP501 = biosimilar adalimumab; ADA = adalimumab; ANBAI = AnBaiNuo (biosimilar adalimumab); BAR_4 = 4mg baricitinib; CERTO = certolizumab pegol; csDMARD = conventional synthetic disease-modifying anti-rheumatic drug; CT-P13 = biosimilar of infliximab; ETN=etanercept; GOL = golimumab; HCQ = hydroxychloroquine; HD203 = etanercept biosimilar; INF = infliximab; IV = intravenous; MTX = methotrexate; RIT = rituximab; SAR_200 = 200mg sarilumab; SB2= biosimilar infliximab 3mg/kg; SB4 = biosimilar etanercept 50mg; SB5=biosimilar adalimumab; SC = subcutaneous; SSZ = sulfasalazine; STD = standard dose; TOC_4 = tocilizumab 4mg/kg; TOC_8 = 8mg/kg tocilizumab; TOF = tofacitinib; ZRC-3197 = biosimilar of adalimumab.

**ACR50 response rate of control group, placebo + MTX (ie, MTX) across interventions and studies (including biosimilars)**


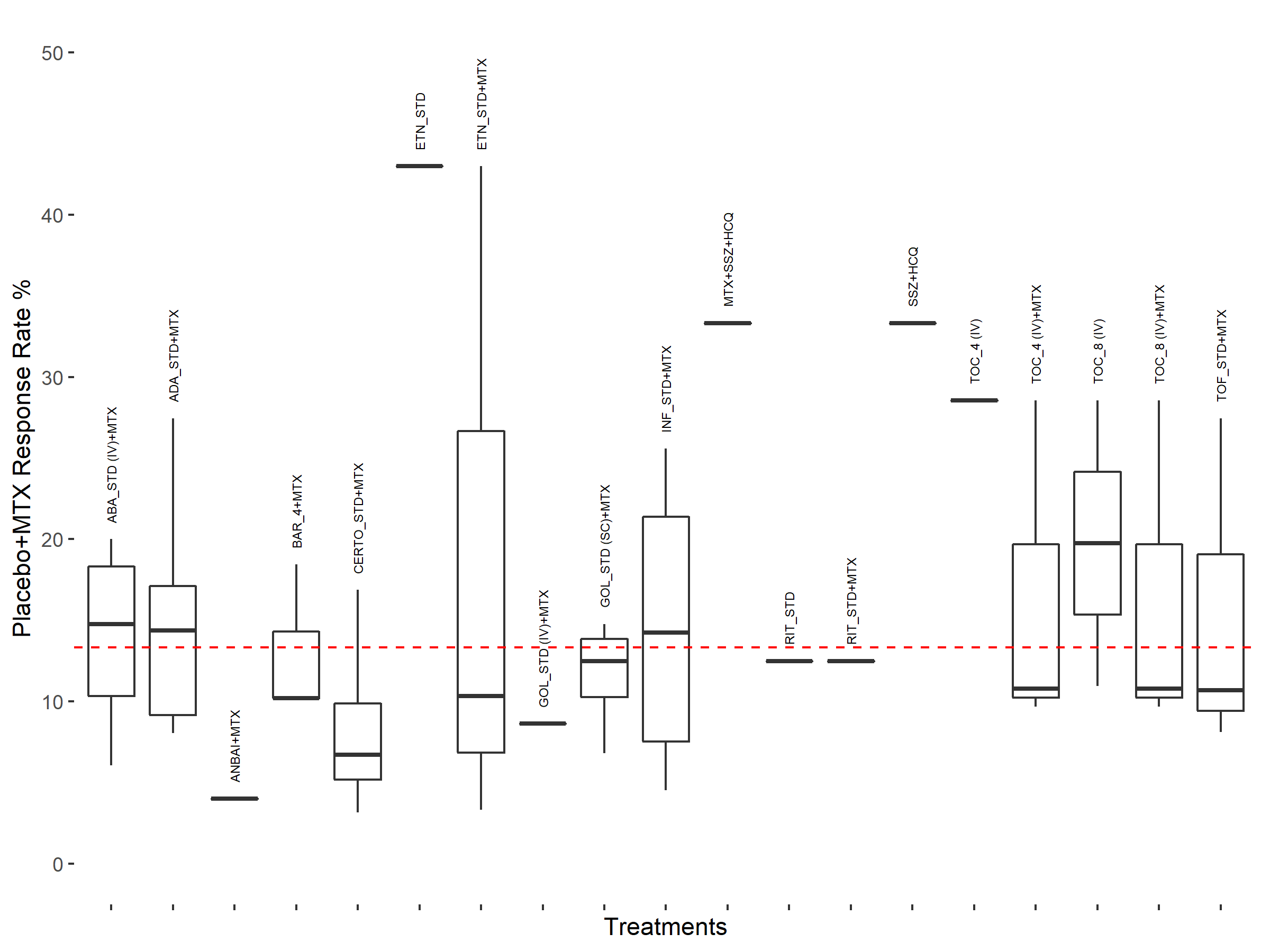


ABA = abatacept; ABP501 = biosimilar adalimumab; ADA = adalimumab; ANBAI = AnBaiNuo (biosimilar adalimumab); BAR_4 = 4mg baricitinib; CERTO = certolizumab pegol; csDMARD = conventional synthetic disease-modifying anti-rheumatic drug; CT-P13 = biosimilar of infliximab; ETN=etanercept; GOL = golimumab; HCQ = hydroxychloroquine; HD203 = etanercept biosimilar; INF = infliximab; IV = intravenous; MTX = methotrexate; RIT = rituximab; SAR_200 = 200mg sarilumab; SB2= biosimilar infliximab 3mg/kg; SB4 = biosimilar etanercept 50mg; SB5=biosimilar adalimumab; SC = subcutaneous; SSZ = sulfasalazine; STD = standard dose; TOC_4 = tocilizumab 4mg/kg; TOC_8 = 8mg/kg tocilizumab; TOF = tofacitinib; ZRC-3197 = biosimilar of adalimumab.

**S**catterplot of placebo response rates versus log (OR) for ACR 50 response (including biosimilars)


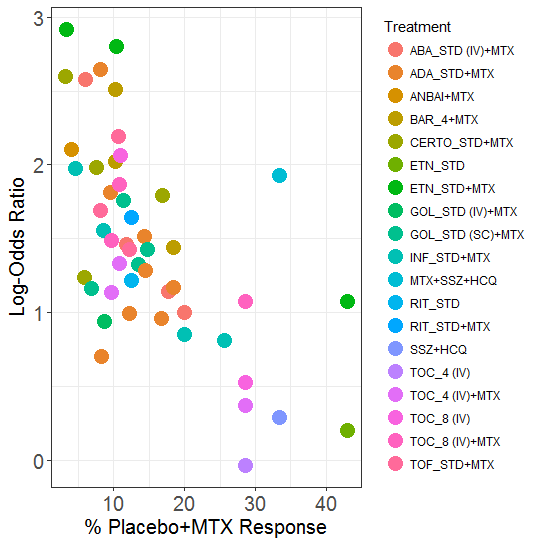


ABA = abatacept; ABP501 = biosimilar adalimumab; ADA = adalimumab; ANBAI = AnBaiNuo (biosimilar adalimumab); BAR_4 = 4mg baricitinib; CERTO = certolizumab pegol; csDMARD = conventional synthetic disease-modifying anti-rheumatic drug; CT-P13 = biosimilar of infliximab; ETN=etanercept; GOL = golimumab; HCQ = hydroxychloroquine; HD203 = etanercept biosimilar; INF = infliximab; IV = intravenous; MTX = methotrexate; RIT = rituximab; SAR_200 = 200mg sarilumab; SB2= biosimilar infliximab 3mg/kg; SB4 = biosimilar etanercept 50mg; SB5=biosimilar adalimumab; SC = subcutaneous; SSZ = sulfasalazine; STD = standard dose; TOC_4 = tocilizumab 4mg/kg; TOC_8 = 8mg/kg tocilizumab; TOF = tofacitinib; ZRC-3197 = biosimilar of adalimumab.

**Summary of Model Fit Information from Unadjusted and Adjusted NMA Models**

**(Including Biosimilars)**

| **Parameter** | **Unadjusted NMA** | **Baseline-risk Adjusted NMA** |
| --- | --- | --- |
| Beta (95% CrI) | NA | -0.66 (-0.87 to -0.43) |
| Heterogeneity (SD, 95% CrI) | 0.34 (0.19 to 0.53) | 0.29 (0.16 to 0.43) |
| Total residual deviance (versus 138 data points | 141.40 | 141.08 |
| DIC | 907.51 | 910.24 |

**Forest Plot of ACR50 Treatment Effect Estimates (OR and 95% CrI) from Unadjusted and Adjusted NMAs (Including biosimilars)**


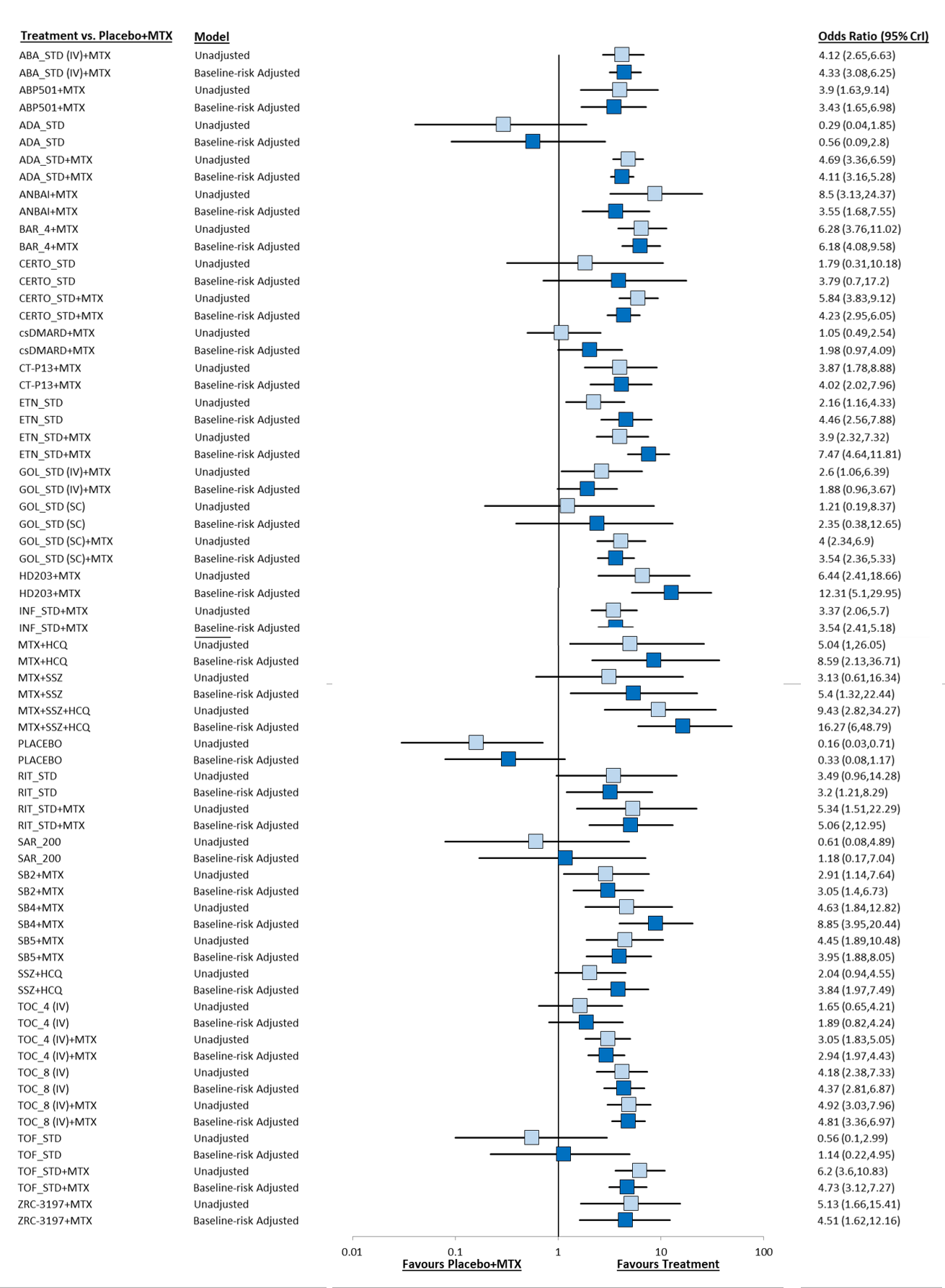


Odds ratios estimated from the unadjusted and control group risk adjusted RE NMAs are presented above, focused on comparisons of active interventions versus placebo. Values are reported with corresponding 95% credible intervals. Odds ratios and >1 favor the active comparator. Downward and upward shifts in treatment effects between NMA approaches can be seen.

**Legend**: ABA = abatacept; ABP501 = biosimilar adalimumab; ADA = adalimumab; ANBAI = AnBaiNuo (biosimilar adalimumab); BAR_4 = 4mg baricitinib; CERTO = certolizumab pegol; csDMARD = conventional synthetic disease-modifying anti-rheumatic drug; CT-P13 = biosimilar of infliximab; ETN=etanercept; GOL = golimumab; HCQ = hydroxychloroquine; HD203 = etanercept biosimilar; INF = infliximab; IV = intravenous; MTX = methotrexate; RIT = rituximab; SAR_200 = 200mg sarilumab; SB2= biosimilar infliximab 3mg/kg; SB4 = biosimilar etanercept 50mg; SB5=biosimilar adalimumab; SC = subcutaneous; SSZ = sulfasalazine; STD = standard dose; TOC_4 = tocilizumab 4mg/kg; TOC_8 = 8mg/kg tocilizumab; TOF = tofacitinib; ZRC-3197 = biosimilar of adalimumab.
